# Helminth diversity of nutria (*Myocastor coypus*) across the Morava basin in the Czech Republic

**DOI:** 10.1017/S0031182024001628

**Published:** 2025-01

**Authors:** Michal Benovics, Eva Nosková, Anna Klimešová, Lucie Škorpíková, Ema Jaššová, Jakub Drimaj, Jan Slováček, Ondřej Mikulka

**Affiliations:** 1 Department of Botany and Zoology, Faculty of Science, Masaryk University, Brno, Czech Republic; 2 Department of Zoology, Faculty of Natural Sciences, Comenius University in Bratislava, Bratislava, Slovakia; 3 Institute of Vertebrate Biology, Czech Academy of Sciences, Brno, Czech Republic; 4 Department of Forest Protection and Wildlife Management, Faculty of Forestry and Wood Technology, Mendel University in Brno, Brno, Czech Republic; 5 Department of Food Technology, Faculty of AgriSciences, Mendel University in Brno, Brno, Czech Republic; 6 Forestry and Game Management Research Institute, Jíloviště, Czech Republic

**Keywords:** biological invasions, coypu, *Echinococcus multilocularis*, parasites, *Strongyloides*, Trichostrongylidae

## Abstract

The nutria was introduced to Europe from South America and kept for the fur industry. This semiaquatic rodent became a well-established species in the Czech Republic; however, it still poses a significant threat to the native fauna, not only as a natural competitor but also as a vector of non-indigenous parasites. Our research aimed to investigate the diversity of endoparasitic helminths in nutria, with a particular focus on assessing the risk posed by helminth species with zoonotic potential. A total of 46 nutria cadavers were collected at 8 locations in the Morava River basin and examined using standard parasitological post-mortem procedures. Additionally, coprological and molecular methods were used to identify the parasites. The presence of 6 helminth species was revealed. The highest prevalence was observed for *Strongyloides myopotami* (78.3%) and *Trichuris myocastoris* (37.0%), both of which are host-specific nematodes of nutria. Only 2 trematode taxa were recorded (*Echinostoma* sp. and a representative of the family Psilostomidae). The presence of alveolar hydatid cysts of *Echinococcus multilocularis* in the livers of 5 nutria specimens was also recorded. Herein, we provide novel molecular data for each parasite species collected, which is valuable for future phylogenetic analyses. Our findings also demonstrate that nutria in the Czech Republic serve as a carrier of helminths with zoonotic potential, particularly *E. multilocularis* and *S. myopotami*. Although the nutria is a relatively new species in local fauna, its synanthropic behaviour raises concerns about potential threats to human health, underscoring the importance of exercising caution when handling these animals.

## Graphical abstract

**Figure:**
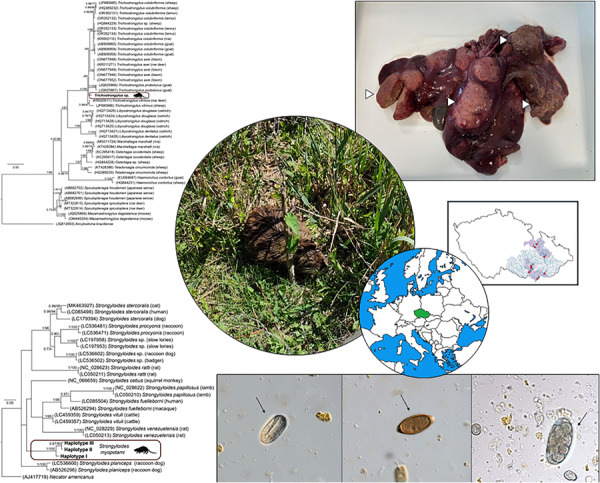

## Introduction

Invasive species represent a pervasive and formidable consequence of globalization, resulting in significant economic damage and posing substantial threats to indigenous biodiversity (Gethöffer and Siebert, 2020). Through their disruptive effects on ecosystems, these species cause declines in biodiversity and may precipitate species extinctions *via* direct predatory activities (Atkinson, 1996). Additionally, they frequently act as vectors for zoonotic diseases or serve as reservoir hosts for such pathogens.

*Myocastor coypus*, commonly known as the nutria or coypu, is prominently featured among the 100 most detrimental invasive species worldwide (Lowe *et al*., 2000) and is formally recognized within the Catalogue of Invasive Alien Species of the European Union (European Commission, 2022). This semi-aquatic rodent, native to South America, is currently distributed across all continents except Australia (and New Zealand) and Antarctica (Woods *et al*., 1992).

The introduction of nutria to the Czech Republic during the early 20th century, primarily driven by fur trading (Anděra, 2011), typifies its successful establishment beyond its native range. Currently, nutrias occupy more than two-thirds of the country's territory (AOPK ČR, 2023). Although there is no general overpopulation, in urban areas where residents feed nutria during winter months, their population size is gradually growing. This trend is further evidenced by a significant increase in official hunting records in recent years: around 1000 individuals in 2010, more than 5000 in 2015, and a staggering 12 580 in 2021 (CZSO, 2024). However, the actual numbers could be several times higher as a large proportion of hunters do not report their catches.

In the Czech Republic, the nutria was initially considered a non-native species with no apparent impact on nature. However, when overpopulated, it damages aquatic and wetland ecosystems to a significant degree, primarily changing the species composition of plants and reducing green biomass (Grace and Ford, 1996; Evers *et al*., 1998; Shaffer *et al*., 2015). With its invasion, nutria can push native species out of the landscape. It also brings the risk of introducing diseases such as tularaemia, brucellosis, leptospirosis or toxoplasmosis to domestic species (Scheuring, 1990; Howerth *et al*., 1994; Bounds *et al*., 2003; Martino *et al*., 2014).

Parasites introduced alongside their hosts into novel territories can profoundly alter natural host–parasite dynamics, thus posing health hazards to native biota and gradually influencing the demography of sympatrically coexisting native fauna (Prenter *et al*., 2004; Dunn, 2009; Britton, 2012). The nutria is host to a relatively high number of parasitic species, with some of them having a zoonotic potential. In its native range, the nutria can harbour more than 30 different helminth species (see Martino *et al*., 2012; Fugassa, 2020). Although the helminth communities in introduced areas are slightly altered, the majority of the parasitic taxa overlap with those from native range populations. However, some parasitic species were recorded only in the non-native range [e.g. *Trichostrongylus duretteae* (Zanzani *et al*., 2016) and *Taenia taeniaeformis* (Umhang *et al*., 2013)].

One representative of zoonotic species, which was previously recorded only within non-native nutria populations, is *Echinococcus multilocularis* [i.e. in France, Germany and Slovenia (Oksanen *et al*., 2016; Romig and Wassermann, 2024)]. Although information on echinococcosis (a disease caused by *Echinococcus* tapeworms) in nutria is limited, a study in western Germany found that feral nutrias are susceptible to *E. multilocularis* infection, but less so than muskrats from the same habitat (Hartel *et al*., 2004). In a French zoo, a captive-born nutria was found to have echinococcosis, presumably introduced through the feces of free-roaming foxes (Umhang *et al*., 2013). Even though this species was not previously recorded from nutria in the Czech Republic, it is a common parasite of foxes in the region, which serve as definitive hosts, and in some counties the prevalence among free roaming foxes reaches more than 50% (i.e. Kolářová *et al*., 1996; Pavlásek 1998; Martínek *et al*., 2001, and data provided by the State Veterinary Administration of the Czech Republic from their previous screenings in 2011). The other species with zoonotic potential is *Strongyloides myopotami*, a specialist of the nutria that causes cutaneous infections in humans known as ‘nutria itch’. Although they do not cause true strongyloidiasis, repeated exposure to larvae of this species may cause an outbreak of severe dermatitis (Little, 1965). This species was previously documented in nutria in the Czech Republic only among farm-bred animals (Nechybová *et al*., 2018).

Information on the parasites of nutria in the non-native range (i.e. Europe) is still rather scarce. Previous studies suggest that the species diversity in this range is lower in comparison with the native range (e.g. Lewis and Ball, 1984; Nardoni *et al*., 2011; Umhang *et al*., 2013; Zanzani *et al*., 2016; Kellnerová *et al*., 2017; Nechybová *et al*., 2018; Ježková *et al*., 2021). While the nutria has retained some of the specialist species from its native range [e.g. *Trichuris myocastoris* (Rylková *et al*., 2015) or *S. myopotami* (Zanzani *et al*., 2016)], it has also acquired some new, often generalist, parasite taxa in non-native areas [e.g. *Trichostrongylus durettae* (Zanzani *et al*., 2016) or *E. multilocularis* (Romig and Wassermann, 2024)]. Nonetheless, there is little comprehensive information on the parasites of free-ranging nutria in the Czech Republic (i.e. Nechybová *et al*., 2018; Ježková *et al*., 2021). In order to compare the diversity of helminth species harboured by Czech nutrias with populations in other countries, the aims of the present study were: (1) to collect data on helminth parasites from different nutria populations in the Czech Republic, (2) to compare the compositions of parasite communities between these populations, and lastly (3) to acquire molecular data for each of the collected helminth species to elucidate genetic structure and intraspecific variability among populations of parasites.

## Material and methods

### Study area and specimen collection

**Figure 1.**
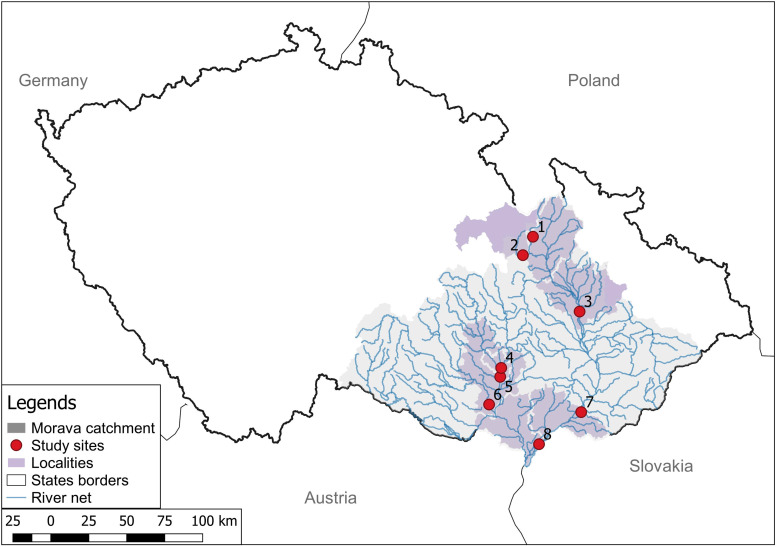
Locations of the sites within the Czech Republic where the investigated nutria individuals were collected. (1) Šumperk; (2) Ústí nad Orlicí; (3) Olomouc; (4) Brno-city; (5, 6) Brno-vicinity; (7) Hodonín; (8) Břeclav.

From January to March 2022, a total of 46 nutria were collected at 8 locations within the Morava catchment in the Czech Republic (Fig. 1, Table 1). The retrieved individuals were placed in plastic bags, and their location, sex and weight were recorded. After transportation to the necropsy room, the viscera were excised and stored frozen.

**Table 1.** List of collection sites and number of processed nutria individuals

Site ID	Site coordinates	Locality	District	N of nutria	M/F ratio
1	49.9832700N, 16.7664256E	Šumperk	Olomouc	5	1/4
2	49.8593331N, 16.7031469E	Ústí nad Orlicí	Pardubice	3	1/2
3	49.5484928N, 17.2717108E	Olomouc	Olomouc	5	5/0
4	49.1656986N, 16.6231097E	Brno-city	South Moravian	2	*/1
5	49.1298936N, 16.6161361E	Brno-vicinity	South Moravian	15	13/2
6	48.9228814N, 16.5600242E	Brno-vicinity	South Moravian	5	2/3
7	48.9735006N, 17.3723258E	Hodonín	South Moravian	5	4/1
8	48.7349381N, 16.9997347E	Břeclav	South Moravian	6	4/2

An asterisk at the sex ratio represents the unidentifiable sex of the specimen.

### Parasite sampling, fixation and identification

Before parasitological examination, the viscera were gradually thawed. The lungs and liver were examined as squash preparations under an SZX7 stereomicroscope (Olympus, Japan). The heart was cut open using scissors and examined macroscopically. The stomach, small intestine, caecum and colon were longitudinally incised, and the contents of the individual organs were separately diluted with water, homogenized and cleaned using a sieve system with the smallest mesh size of 150 μm. Subsequently, the material retained on the sieve, as well as the walls of the individual organs, was examined under the stereomicroscope.

The collected helminths were counted and stored in 70% ethanol for further morphological analyses. When possible, at least 5 individuals of each helminth species per host specimen were preserved in 96% pure-grade ethanol for DNA extraction. Before fixation in Canada balsam, digeneans were stained in iron acetocarmine, following the protocol of Georgiev *et al*. (1986). The species identification of digeneans and selection of measurements for morphometric comparison followed the procedures outlined by Gibson *et al*. (2002), Jones *et al*. (2005) and Bray *et al*. (2008). Nematodes were mounted on slides, covered in a mixture of glycerine and water (in a ratio of 3:7), and cleared by gradually increasing the volume of glycerol, according to Moravec (2013).

To support the findings from the parasitological necropsy, fresh fecal samples available from 20 out of all examined individuals were subjected to coproscopical examination using the modified Sheather's sugar flotation method (Sheather, 1923; Jirků-Pomajbíková and Hůzová, 2018). Briefly, walnut-sized fecal samples were homogenised with water using a mortar and pestle, sieved into a tube and centrifuged at 2000 rpm for 3 min. The supernatant was removed, and the sediment was mixed with a sugar solution with a density of 1.3 g/cm^3^. The sample was centrifuged again at 2000 rpm for 3 min. A surface film containing parasite stages was transferred to a slide with an inoculating loop, covered with a coverslip and examined under a light microscope (Foreyt, 2002).

Primary epidemiological data, including prevalence, mean abundance and minimum and maximum intensities of infection, were calculated for each parasite species according to Bush *et al*. (1997). Prevalence was defined as the percentage of host individuals infected by a given parasite species, and mean abundance was calculated as the mean number of parasite specimens per individual host considering both infected and uninfected hosts. Following the suggestion of Rózsa *et al*. (2000) for interpreting epidemiological data, a confidence interval at the level of 95% was calculated for mean abundance.

### PCR amplification, sequence analysis and phylogenetic analyses

Total DNA from fresh or frozen fecal samples (*N* = 40) was isolated using PowerSoil DNA isolation kit (Qiagen Company, USA). The extracted fecal DNA was screened by qPCR for the detection of *Strongyloides* (18S rDNA) using a Real-Time PCR LightCycler^®^ 480 (Roche, Switzerland). The primers and reaction conditions used followed Verweij *et al*. (2009).

Parasite genomic DNA was extracted using NucleoSpin^®^ Tissue kit (Macherey-Nagel, Düren, Germany) following the manufacturer's protocol. Prior to extraction, the specimens (or their parts) were removed from the ethanol and dried in a thermal block. The PCR reactions were performed in a 20 μL reaction mixture containing 14 μL nuclease-free water, 4 μL FIREPol Master Mix Ready to Load (Solis BioDyne, Tartu, Estonia), 0.5 μm of each primer and 1 μL of DNA template. PCR products were detected by electrophoresis in 1% agarose gels stained with GoodView (SBS Genetech, Bratislava, Slovakia). A total of 8 different primer combinations were used for the amplification of specific genomic regions in nematodes, trematodes and cestodes. A list of primer sequences and cycling conditions is provided in Table 2. The resulting amplicons were subsequently subjected to Sanger sequencing, after which the obtained sequences were compared *in silico* with data available in publicly accessible databases and were also used for phylogenetic analyses.

**Table 2.** List of primers used for PCR amplification of mitochondrial and nuclear markers in the present study

Target taxa	Region	Primer name	Direction	Sequence (5′-3′)	PCR thermal profile	Reference
*Strongyloides* spp.	18S rDNA	Nem_18S_F	Forward	CGCGAATRGCTCATTACAACAGC	94°C, 5 min; 35 × (94°C, 30 s; **54°C, 30 s**; 72°C, 60 s); 72°C, 10 min	Floyd *et al*. (2005)
		Nem_18S_R	Reverse	GGGCGGTATCTGATCGCC		
*Strongyloides* spp., *Trichostrongylus* spp.	COI mtDNA	SPP_COX_F	Forward	TTTGATCCTAGTTCTGGTGGTAATCC	94°C, 10 min; 45 × (94°C, 10 s; **60°C, 10 s**; 72°C, 10 s); 72°C, 2 min	Barratt *et al*. (2019)
		SPP_COX_R	Reverse	GTAGCAGCAGTAAAATAAGCACGAGA		
*Trichostrongylus* spp.	ITS1-5.8S-ITS2 rDNA	NC5	Forward	GTAGGTGAACCTGCGGAAGGATCATT	94°C, 10 min; 30 × (94°C, 30 s; **55°C, 30 s**; 72°C, 30 s); 72°C, 10 min	Newton *et al*. (1998)
		NC2	Reverse	TTAGTTTCTTTTCCTCCGCT		
*Trichuris* spp.	ITS1-5.8S-ITS2 rDNA	NC5	Forward	GTAGGTGAACCTGCGGAAGGATCATT	94°C, 10 min; 35 × (94°C, 60 s; **55°C, 60 s**; 72°C, 60 s); 72°C, 10 min	Rylková *et al*. (2015)
		NC2	Reverse	TTAGTTTCTTTTCCTCCGCT		
Digenea	COI mtDNA	JB3	Forward	TTTTTTGGGCATCCTGAGGTTTAT	95˚C, 2 min; 40 × (95°C, 30 s; **50°C, 30 s**; 72°C, 60 s); 72°C, 2 min	Bowles & McManus (1993); Miura *et al*. (2005); Benovics *et al*. (2022)
		COI-R_trema	Reverse	CAACAAATCATGATGCAAAAGG		
Digenea	28S rDNA	LSU5	Forward	TAGGTCGACCCGCTGAAYTTAAGCA	94˚C, 3 min; 40 × (94°C, 30 s; **56°C, 30 s**; 72°C, 2 min); 72°C, 7 min	Olson *et al*. (2003)
		1500R	Reverse	GCTATCCTGAGGGAAACTTCG		
*Echinococcus* spp.	16S mtDNA	rrnS-F	Forward	AGCCAGGTCGGTTCTTATCTATTG	94°C, 10 min; 35 × (94°C, 60 s; **61°C, 30 s**; 72°C, 60 s); 72°C, 10 min	Šoba *et al*. (2020)
		rrnS-R	Reverse	CGAGGGTGACGGGCGGTGTGTAC		
*Echinococcus* spp.	NAD5 mtDNA	NAD5f	Forward	GCCCCIACICCAGTIAGTTCT	94°C, 10 min; 35 × (94°C, 60 s; **50°C, 30 s**; 72°C, 60 s); 72°C, 10 min	Šoba *et al*. (2020)
		NAD5r	Reverse	AAIACACTTAGAIACICCATGACT		

In order to assess the phylogenetic position of selected parasite taxa (i.e. in the case of strongylids) and/or selected haplotypes (i.e. in the case of COI variants of various *Strongyloides* spp.), additional orthologous sequences from congeners or different phylogenetically close species were obtained from GenBank (accession numbers are included within phylogenetic trees). The sequences were aligned by means of the fast Fourier transform algorithm implemented in MAFFT (Katoh *et al*., 2002), using the G-INS-i refinement method. A general time-reversible model (GTR; Lanave *et al*., 1984) was applied for each partition of the alignment, each representing an individual genomic region. For the *Strongyloides* spp. dataset, the GTR model was applied for the entire length of the alignment. Phylogenetic trees were constructed using Bayesian inference (BI) and maximum likelihood (ML) approaches in MrBayes 3.2. (Ronquist *et al*., 2012) and RAxML 8.1.12 (Stamatakis, 2006, 2014), respectively. The BI analysis used the Metropolis-coupled Markov chain Monte Carlo algorithm with 2 parallel runs of 1 cold and 3 hot chains, and was run for 10^6^ generations, sampling trees every 100 generations. The initial 30% of all saved trees were discarded as ‘burn-in’ after checking that the standard deviation split frequency fell below 0.01. The convergence of the runs and the parameters of individual runs were checked using Tracer v. 1.7.1 (Rambaut *et al*., 2018). Posterior probabilities for each tree node were calculated as the frequency of samples recovering a given clade. The clade bootstrap support for ML trees was assessed by simulating 10^3^ pseudoreplicates.

## Results

### Nutria's helminth diversity and epidemiology

**Figure 2.**
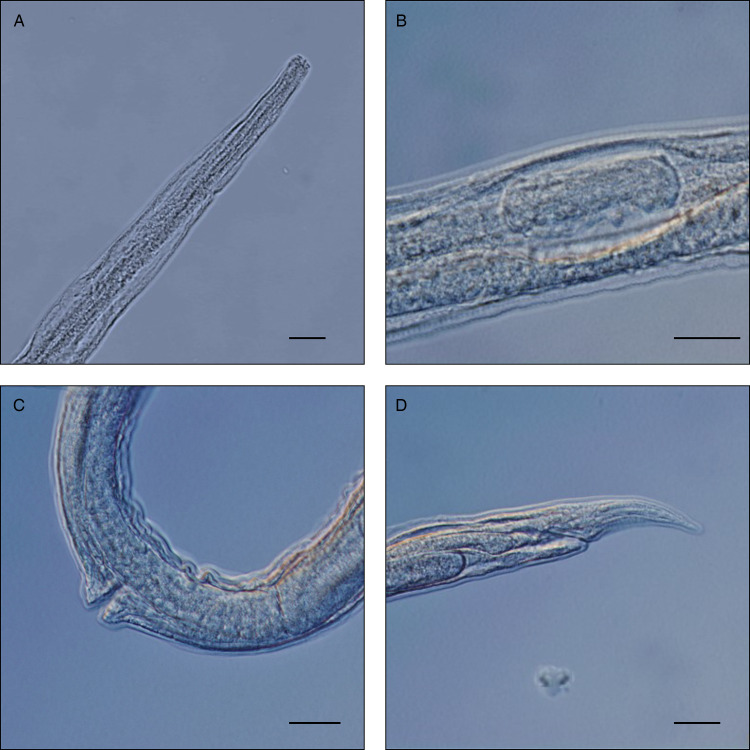
Microscopic details of *Strongyloides myopotami* parasitic females. Scale bar = 20 μm. (A) anterior end; (B) egg in the uterus; (C) detail of vulva; (D) posterior end with tail and anus.

**Figure 3.**
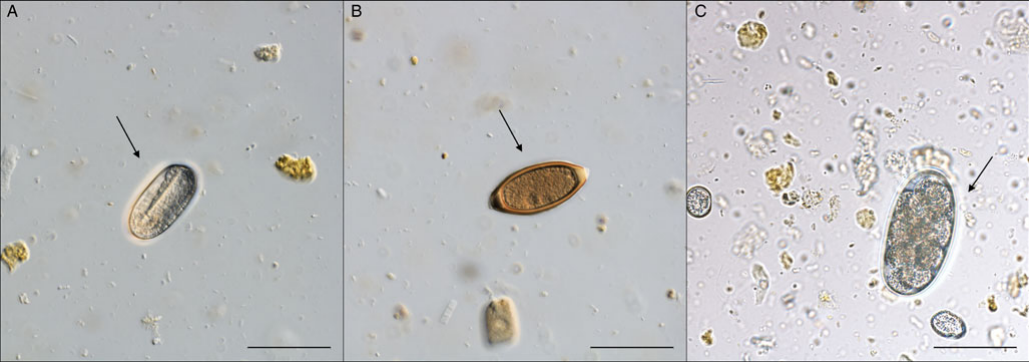
Egg stages of Nematoda detected in nutria fecal samples using Sheather's sugar flotation. Scale bar = 50 μm. (A) Oval egg with so-called U-larva of *Strongyloides myopotami*; (B) egg of *Trichuris myocastoris*; (C) egg of strongylid nematode, probably *Trichostrongylus* sp.

**Figure 4.**
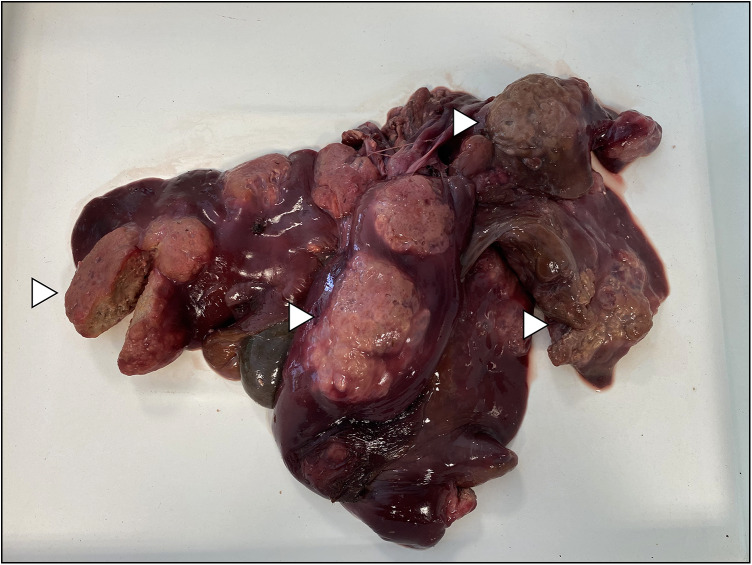
Alveolar hydatid cysts of *Echinococcus multilocularis* in the liver of nutria collected near the city of Šumperk. Examples of cysts are pinpointed by white arrows.

**Figure 5.**
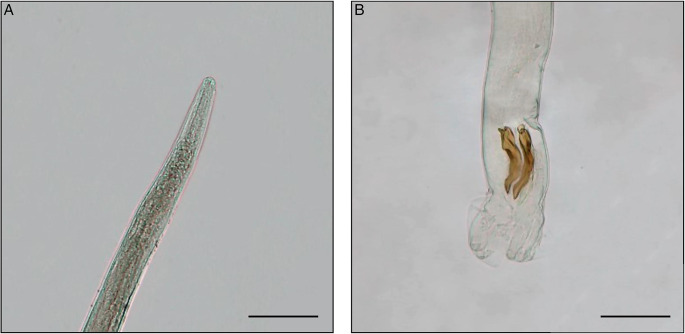
Microscopic details of *Trichostrongylus* sp. specimen. Scale bar = 100 μm. (A) The anterior end of the male; (B) the posterior end of the male with spicules and copulatory bursa.

A total of 6 helminth species were collected from 46 nutria specimens during microscopical examination (Table 3). The highest prevalence (67.4%) was recorded for *S. myopotami*, which parasitized nutrias from all collection sites (Fig. 2). This species also exhibited the highest mean abundance and maximum intensity of infection among the examined individuals. The flotation method revealed this species (as infectious eggs) in each fresh fecal sample (Fig. 3), even when the examination of the viscera did not yield any adult specimens. After combining the data from necropsy and flotation, the overall prevalence increased to 78.3%. In addition, qPCR analysis confirmed the presence of *Strongyloides* DNA in 100.0% of the tested fecal samples, further corroborating its high prevalence. Combining all 3 methods, the total prevalence of *S. myopotami* reached 93.5% among all processed nutria individuals. The second most prevalent species was *T. myocastoris*, which was, during dissection, recorded in nutria from all sites except Olomouc (site 3) and Břeclav (site 8). Molecular analysis revealed that this species was genetically identical in the ITS1-5.8S-ITS2 region to *T. myocastoris* specimens previously documented in the Czech Republic (MF077367). *Echinococcus multilocularis* hydatids (Fig. 4) were found in the livers of 5 individuals, altogether from 3 sites (Table 3). A further unidentified trematode species was also found in 5 nutria individuals (collected from 2 nutrias from site 6, 2 nutrias from site 8 and 1 nutria from site 3). Due to the poor quality of the material, especially as a result of deep freezing the cadavers, morphological identification of these trematodes was not possible. However, the molecular sequences (partial 28S rDNA and partial COI mtDNA regions) suggest that these specimens belonged to the family Psilostomidae, as they had 99.8% similarity to a sequence of Psilostomidae gen. sp. in GenBank (MN726950; see Table 3 with accession numbers for the newly obtained DNA sequences). *Trichostrongylus* nematodes were also recorded in nutria from 3 collection sites, but could not be identified to species level due to the insufficient number and quality of specimens for morphological evaluation (Fig. 5). Additionally, a single nutria individual, collected in the vicinity of Brno city (site 6), was parasitized by *Echinostoma* trematodes. Only 2 specimens of this helminth species were collected and all visible morphological features resembled *Echinostoma revolutum*.

**Table 3.** List of collected parasite species identified during microscopical examination, with localization, basic epidemiological data, sites with positive records, GenBank accession numbers to newly generated sequences and information about previous records from nutria in the Czech Republic

Species	Localization	*P*	*A*	*I*	Sites	GB no.	Previous records in Czech Republic (prevalence)	Reference
*Strongyloides myopotami*	Small intestine, large intestine	67.4% (93.5%)	21.89 ± 16.29	1–206	All	PQ568308–PQ568310 (COI); PQ568420 (18S)	Farm-bred nutria (11.5%), feral nutria (25.0–30.0%)	Nechybová *et al*. (2018)
*Trichuris myocastoris*	Caecum, large intestine, small intestine	37.0% (39.1%)	3.26 ± 3.37	1–37	1, 2, 4, 5, 6, 7 (3)	PQ571257, PQ571258 (ITS)	Farm-bred nutria (57.0%), feral nutria (5.0–40.0%)	Nechybová *et al*. (2018)
*Trichostrongylus* sp.	Small intestine	8.7% (10.8%)	0.43 ± 0.80	3–9	3, 5 (6)	PQ567056–PQ567059 (COI); PQ570797 (ITS)	Farm-bred nutria (4.0%)	Nechybová *et al*. (2018)
*Echinostoma* sp.	Small intestine	2.2%	0.04 ± 0.00	2	6	–	N/A	N/A
Psilostomidae gen. sp.	Small intestine	10.8%	0.21 ± 0.30	1–3	3, 6, 8	PQ569938 (28S)	N/A	N/A
*Echinococcus multilocularis*	Liver	10.8%	–	–	1, 2, 5	PQ786491 (NAD5)	N/A	N/A

*P* = prevalence; *A* = mean abundance with confidence interval at the level of 0.95; *I* = intensity of infection (min–max); GB no. = GenBank accession numbers to the representative sequences. Prevalence values and site numbers in brackets are for records including flotation and quantitative PCR results. Dashes (–) indicate the species was recorded in an uncountable number of cysts. N/A indicates that this parasite taxon was not previously recorded in nutria from the Czech Republic.

### Genetic diversity and variability in obtained nematode species

All obtained helminth species were sequenced to analyse genetic diversity and intraspecific variability. For trematodes, no intraspecific variability was observed in either the 28S or COI region. For *T. myocastoris* and *Trichostrongylus* sp. only a minor intraspecific variability was observed in COI and no variability in ITS regions. A total of 49 *S. myopotami* specimens were sequenced, all of which were identical in the 18S region to the *S. myopotami* sequence deposited in GenBank (AB453313). The amplified COI region spanned 240 nucleotide positions, and 3 distinct genetic variants were identified among these individuals. Haplotype I was observed in individuals at all sites where *S. myopotami* was present. Haplotype II was absent in individuals from Šumperk (site 1). All 3 haplotypes were found only in individuals from the Brno-vicinity (site 6), with only 1 sequenced individual carrying haplotype III. Haplotypes II and III were the most similar, differentiating in only 1 substitution. Only a single *S. myopotami* specimen was collected from Ústí nad Orlicí; however, PCR amplification did not yield products of sufficient quality, and therefore, it was not possible to assess the haplotype present at this site.

**Figure 6.**
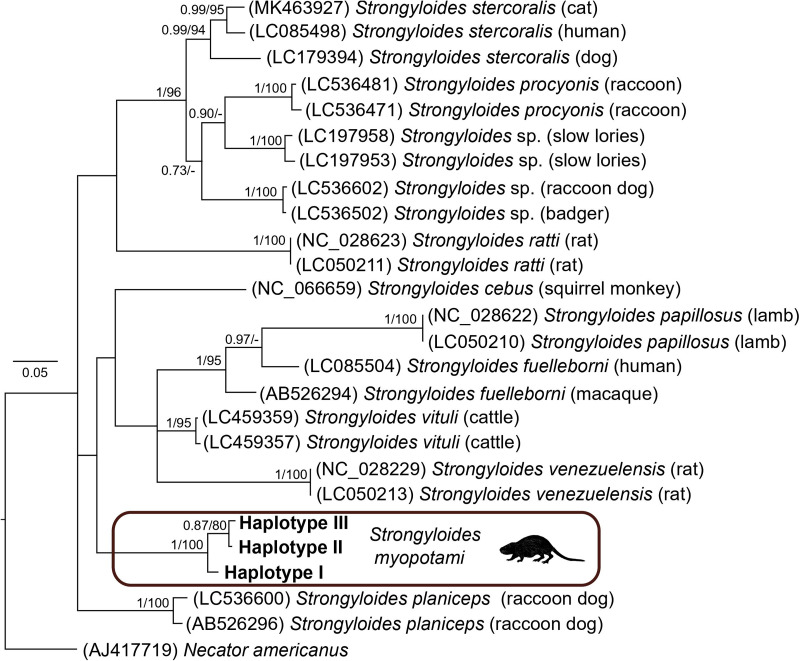
Phylogenetic tree of 25 sequences of *Strongyloides* species reconstructed by Bayesian inference. The tree is rooted using *Necator americanus* as an outgroup. Values at the nodes indicate posterior probabilities from BI and bootstrap values from ML analyses. Dashes indicate nodal support values below 0.70 and 50, respectively. The hosts of respective *Strongyloides* specimens are noted in brackets.

The resulting *Strongyloides* phylogenetic tree revealed 12 separate clades corresponding to different *Strongyloides* species. For the partial COI mtDNA region of *Strongyloides*, the alignment consisted of 26 sequences, including *Necator americanus* as the outgroup. Both BI and ML analyses generated trees with identical topologies. The BI tree with posterior probabilities and bootstrap values (corresponding to the ML tree) along respective nodes is presented in Fig. 6. All the haplotypes of *S*. *myopotami* from nutrias clustered within a highly supported subclade. However, its position was not fully resolved due to the basal polytomy of the tree.

**Figure 7.**
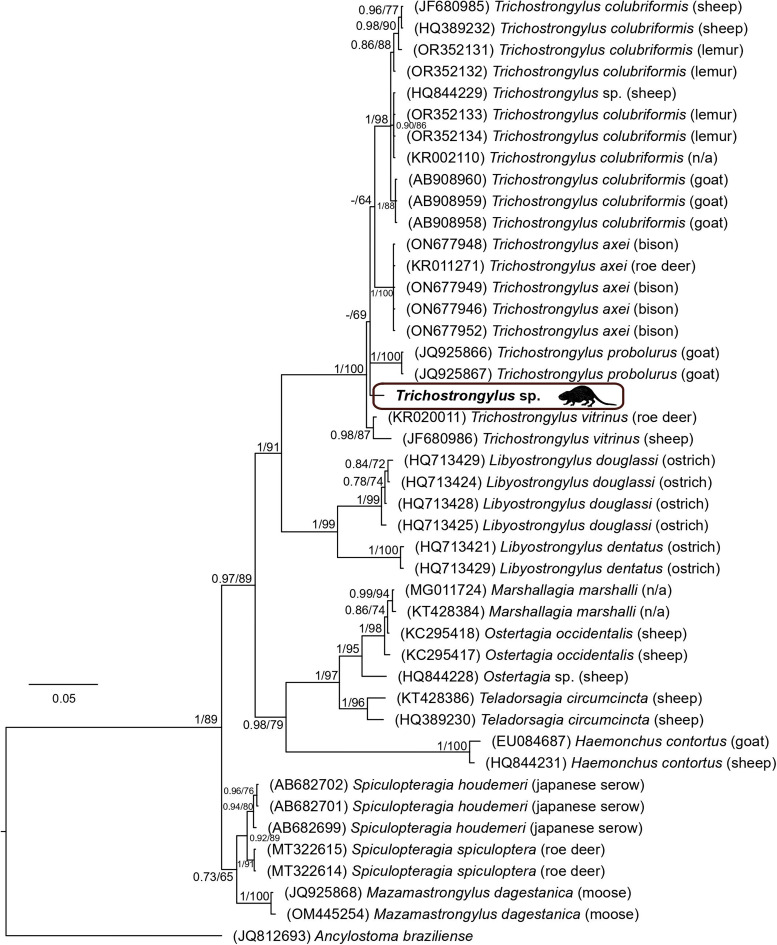
Phylogenetic tree of 43 sequences of 15 trichostrongylid species reconstructed by Bayesian inference. The tree is rooted using *Ancylostoma braziliense* as an outgroup. Values at the nodes indicate posterior probabilities from BI, and bootstrap values from ML analyses. Dashes indicate nodal support values below 0.70 and 50, respectively. The hosts of respective trichostrongylid specimens are noted in brackets. N/A indicates that the host information is not available.

The final sequence alignment, encompassing selected trichostrongylid species and the outgroup *Ancylostoma braziliense*, was constructed using the ITS1-5.8S-ITS2 region. The alignment included 44 taxa and spanned 775 unambiguously aligned nucleotide positions. The BI tree, with posterior probabilities and bootstrap values (corresponding to the ML tree) along respective nodes, is presented in Fig. 7. *Trichostrongylus* spp. formed a well-supported monophyletic group, with the sister position to the *Libyostrongylus* spp. The *Trichostrongylus* sp. collected from nutria in the Czech Republic clustered within the ‘*Trichostrongylus*’ clade; however, its position was not fully resolved, resulting in polytomy. This *Trichostrongylus* sp. shared 98.3% sequence similarity with *T. vitrinus* which was collected from Roe deer (*Capreolus capreolus*) in Russia. The species collected from nutrias in the Czech Republic exhibited greater genetic dissimilarity (>2%) to *T. axei* or *T. colubriformis*, suggesting it represents a distinct species.

The sequencing of 2 mitochondrial genomes supported the identification of the hydatid cysts as *E. multilocularis.* The individuals collected from nutrias in the Czech Republic were genetically identical in the NADH region to those from nutrias in Slovenia (MW560732). In contrast, in the 16S rRNA region, they were identical to specimens from France (e.g. the sequence retrieved from the complete mitochondrial genome; OQ599967) and differed by a single nucleotide substitution from those collected in Slovenia (MW558108).

## Discussion

Since the introduction of nutria to the Czech Republic, its population size has been steadily increasing. Despite its established status as an invasive species in the region, the parasite fauna associated with nutria remains critically understudied. In its native range, 64 parasite taxa have been recorded, which were assigned either to species level or at least to a higher taxonomic level (e.g. El-Kouba *et al*., 2009; Issia *et al*., 2009; Rossin *et al*., 2009; Martino *et al*., 2012; Benati *et al*., 2017; Fugassa, 2020). In contrast, European studies have documented only 11 of these parasites in nutria, with an additional 13 taxa identified exclusively in European nutria populations (Lewis and Ball, 1984; Ménard *et al*., 2001; Nardoni *et al*., 2011; Umhang *et al*., 2013; Zanzani *et al*., 2016; Nechybová *et al*., 2018; Ježková *et al*., 2021). Thus far, 24 species have been identified in Europe, indicating that the diversity of nutria parasites is markedly higher in their native range compared to the regions where they have been introduced.

Information on nutria parasites in the Czech Republic is scarce and only a few studies have provided any comprehensive insight, based on a combination of diagnostic methods. Regarding helminths, only 2 genera were previously recorded; *S. myopotami* was recorded among fur-farmed animals in the Czech Republic with 25% prevalence (out of 20 examined animals, Nechybová *et al*., 2018). In the same study, also a further unidentified *Strongyloides* species was detected by coprological methods in free roaming nutrias at various sites in the Czech Republic, although it is highly possible it will also be *S. myopotami*, in accordance with our study. The other taxon was *T. myocastoris*, which was recorded among farmed animals (Rylková *et al*., 2015; Nechybová *et al*., 2018) and feral animals, as well (Nechybová *et al*., 2018).

On the basis of the necropsy of 46 nutrias, the coproscopy of feces from 20 individuals, the amplification of 40 extracted fecal DNA samples using qPCR (18S rDNA) and sequencing of the 18S ribosomal subunit DNA and partial COI mtDNA, the highest recorded prevalence was of *S. myopotami* (93.5%). When coproscopy and molecular analyses were not considered, the prevalence of this parasite appeared lower. Specifically, the flotation method revealed the presence of this parasite in 5 nutrias, although no adult parasites were found during the necropsy of those individuals. This discrepancy suggests that the infection intensity in these cases may have been too low to be detected during necropsy. In contrast, qPCR analysis, the most sensitive method employed, detected *Strongyloides* in 100% of the analysed fecal samples. Previous studies have also reported high prevalence rates of *S. myopotami*. Choe *et al*. (2014) documented a 100.0% prevalence in a survey of 10 nutrias in Korea. Babero and Lee (1961) recorded a prevalence of 62.5% in 56 nutrias from Louisiana (USA). In the native range of nutria, a prevalence of 26.7% was observed (Martino *et al*., 2012). The highest prevalence in Europe to date was found in Italy, at 63.4% (Zanzani *et al*., 2016). Our current study also ranks among those in which *S. myopotami* was the most commonly represented parasite, and its prevalence among Czech nutria populations appears to be comparatively higher, as previously reported by Nechybová *et al*. (2018) (25–30%).

The second most common parasite was *T. myocastoris* (prevalence 37.0%). Its significance as a common parasite of nutrias is corroborated by Babero and Lee (1961), who recorded prevalences of 28 and 50% at 2 study sites in Louisiana (USA). Similarly, Martino *et al*. (2012) observed *T. myocastoris* among the most numerous parasite species in South America, with a prevalence of 13.8%. In the Czech Republic, Nechybová *et al*. (2018) recorded a prevalence of 40% for *T. myocastoris* in a study of wild nutrias and a prevalence of 5% for *Trichuris* sp. in fecal samples from farmed nutrias.

The genus *Trichostrongylus* could not be definitively identified at the species level. Identification is primarily based on the shape and size of male spicules and copulatory bursa, while females are consistently difficult to identify. According to Dikmans’ (1937) key, the *Trichostrongylus* specimens in our study most closely resembled *T. ransomi* in spicule shape and length. Another distinguishing characteristic was the distance of the anus from the tail tip in females. When compared to the work of Ghasemikhah *et al*. (2011), the spicule shape (resembling a high-heeled shoe) was similar to that in *T. colubriformis* species. Previous studies have reported the presence of *Trichostrongylus duretteae*, *T. sigmodontis*, *T. colubriformis* and *T. retortaeformis* in nutrias (Babero and Lee, 1961; Zanzani *et al*., 2016; Fugassa, 2020). Based on newly generated DNA sequences, our species was genetically most similar to *T. axei*. However, sequences for *T. sigmodontis* or *T. duretteae* are not available in GenBank, so genetic similarity to these species could not be assessed. Our mitochondrial DNA sequences, representing a more variable region, make comparisons with GenBank data less meaningful unless sequences from the same or closely related organisms are available.

The trematode species with a prevalence of 11% was classified within the family Psilostomidae based on the DNA sequence similarity of the large ribosomal subunit (28S). Mitochondrial DNA (COI) did not assist in identifying the parasite, as no closely related sequence was available in the GenBank database. The family Psilostomidae comprises 13 genera, which are gastrointestinal parasites of birds and mammals. Morphologically, the members of this family resemble those of the family Echinostomatidae, but Psilostomidae lack the characteristic spined collar (Kostadinova, 2005; Atopkin, 2011). Given the presence of the genus *Psilotrema* in Europe (Atopkin, 2011) and the closest genetic similarity observed, it is plausible to hypothesize that our specimens belong to this genus. Additionally, *Psilostomum* sp. has also been previously reported in nutrias, although only in the metacercarial stage; however, it was not identified in our study (Babero and Lee, 1961).

The presence of *E. multilocularis* in the native range of nutria remains undocumented. In Europe, the occurrence and increasing incidence of infections with the zoonotic tapeworm *E. multilocularis* are closely linked with the rising populations of foxes, raccoon dogs and nutrias (Janovsky *et al*., 2002; Romig, 2009; Križman *et al*., 2022). Similarly, the spread of this parasite correlates with growing urban fox populations and the movements of infected dogs (Goodfellow *et al*., 2006). Within the Morava basin, the presence of echinococcosis was detected in nutria at the Šumperk (site 1), Ústí nad Orlicí (site 2) and Brno-vicinity (5) locations, with the former 2 being only about 20 km apart. Although *E. multilocularis* was previously recorded among foxes in all 3 respective Czech districts [ranging from 10.0 to 40.0% prevalence, see Kolářová *et al*. (2017) and references therein], this finding substantiates the Eurasian and somewhat disjunct distribution of the parasite (McManus *et al*., 2003; Oksanen *et al*., 2016). Our samples showed a high genetic similarity with *E. multilocularis* from various European countries, particularly Slovenia and France. This genetic congruence classifies the Moravian nutria specimens within the widely distributed genotype observed across western, central and eastern Europe (Santoro *et al*., 2024).

Studies on *E. multilocularis* tend to focus on the occurrence of the parasite in definitive hosts, particularly foxes. The increase in fox populations in western European countries, attributed to rabies vaccination programmes (Eckert *et al*., 2000), probably prompted the migration of young foxes from areas with high population density to those with lower density, moving eastward (Sréter *et al*., 2003). In the 1990s, changes in land use occurred in the former communist countries. Additionally, a decline in fox hunting, driven by a fall in the value of fox fur and the implementation of rabies vaccination programmes in central and eastern European countries, contributed to a rise in the fox population (Szemethy *et al*., 2000). This increase in fox numbers likely led to a higher prevalence and spread of *E. multilocularis* (Sréter *et al*., 2003).

Currently, *E. multilocularis* is widely distributed in Europe (ESFA, 2021). The emergence of a new potential definitive host, the invasive raccoon dog (Sutor, 2008), has also facilitated this spread. This species is now well established in Europe and continues to expand its range westward and southward across the continent (Genovesi, 2009). However, the prevalence of *E. multilocularis* in raccoon dogs is lower compared to foxes, which are still considered the primary definitive host for this parasite.

A study conducted by Oksanen *et al*. (2016) demonstrated that nutrias generally play a minimal or no role in the life cycle of *E. multilocularis* within European Union countries. Studies investigating the presence of *E. multilocularis* in European nutrias found a prevalence of 0.4% from 12 locations in western France (Umhang *et al*., 2013) and 5.9% in 2 distinct areas southwest of North Rhine-Westphalia in Germany (Hartel *et al*., 2004). Nonetheless, in areas with moderate to high infection prevalence in red foxes, nutrias may contribute to the life cycle of this parasite (Oksanen *et al*., 2016). Studies conducted in France and Slovenia have identified nutrias as bioindicators of *E. multilocularis* presence in the environment (Umhang *et al*., 2013; Križman *et al*., 2022). Consequently, it is plausible that foxes near the Šumperk, Ústí nad Orlicí and Brno-vicinity locations could also be infected with *E. multilocularis*.

## Data Availability

The data supporting the conclusions of this study are included in this article. The newly generated sequences were submitted to the GenBank database under accession numbers PQ567056–PQ567059; PQ568308–PQ568310; PQ568420; PQ569938; PQ570797; PQ571257–PQ571258; PQ786491.
